# New Antioxidative Secondary Metabolites from the Fruits of a Beibu Gulf Mangrove, *Avicennia marina*

**DOI:** 10.3390/md12084353

**Published:** 2014-07-29

**Authors:** Cheng-Hai Gao, Xiang-Xi Yi, Wen-Pei Xie, Yin-Ning Chen, Ming-Ben Xu, Zhi-Wei Su, Lian Yu, Ri-Ming Huang

**Affiliations:** 1 Guangxi Key Laboratory of Marine Environmental Science, Guangxi Academy of Sciences, Nanning 530007, China; E-Mails: gaochenghai@gxas.cn (C.-H.G.); xwpei-028@163.com (W.-P.X.); xmben0771@126.com (M.-B.X.); suzw1454@126.com (Z.-W.S.); lianyu@gxu.edu.cn (Y.L.); 2 School of Pharmaceutical Sciences, Guangxi University of Chinese Medicine, Nanning 530001, China; E-Mail: xiangxiyi81@aliyun.com; 3 Key Laboratory of Plant Resources Conservation and Sustainable Utilization, South China Botanical Garden, Chinese Academy of Sciences, Guangzhou 510650, China; E-Mail: chendianyu3356@163.com; 4 Department of Pharmacy and Pharmacology, University of Bath, Bath BA2 7AY, UK

**Keywords:** antioxidant, *Avicennia marina*, caffeoyl glycoside, marinoid, phenylethyl glycoside

## Abstract

Further chemical investigation of the fruits of the mangrove, *Avicennia marina*, afforded three new phenylethyl glycosides, marinoids J–L (**1**–**3**), and a new cinnamoyl glycoside, marinoid M (**4**). The structures of isolates were elucidated on the basis of extensive spectroscopic analysis and by comparison of the data with those of related secondary metabolites. The antioxidant activity of the isolates was evaluated using the cellular antioxidant assay (CAA), and compounds **1**–**4** showed antioxidant activities, with EC_50_ values ranging from 23.0 ± 0.71 μM to 247.8 ± 2.47 μM.

## 1. Introduction

**Figure 1:**
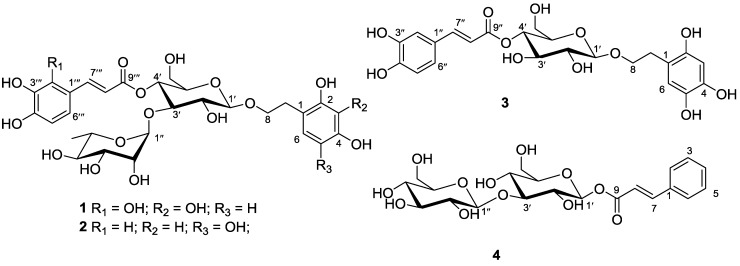
Secondary metabolites **1**–**4**.

During the course of our search for new bioactive secondary metabolites from the mangrove, * Avicennia marina* (Forsk.) Vierh. (Acanthaceae family), some jacaranone analogs, marinoids F–I [1], have been obtained. Because of our interest in searching for new bioactive natural products, the continuing investigation on the chemical constituents of the fruits of this specimen was carried out and resulted in the isolation of three new phenylethyl glycosides, marinoids J–L (**1**–**3**), and a new cinnamoyl glycoside, marinoid M (**4**) (Figure 1). This paper deals with the isolation, structural elucidation and antioxidant activity of these secondary metabolites.

## 2. Results and Discussion

**Figure 2:**
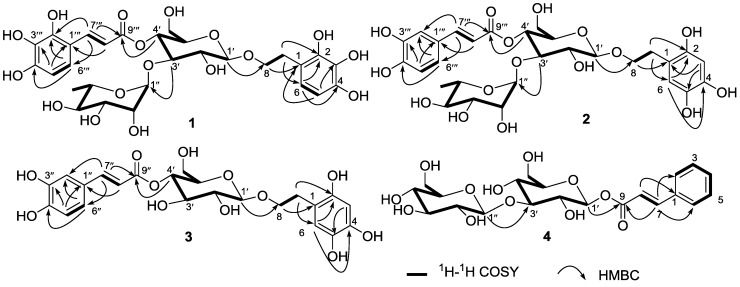
Selected ^1^H–^1^H COSY and HMBC correlations of **1**–**4**.

Marinoid J (**1**), a yellow oil, has a molecular formula identified as C_29_H_36_O_17_ based on the NMR and HRESIMS data ([M + H]^+^, *m*/*z*: 657.2029; calcd. for C_29_H_37_O_17_ *m*/*z*: 657.2031). The ^1^H, ^13^C NMR (Table 1), COSY and HMQC spectra of **1** showed the presence of two AB system signals at δ_H_ 6.71 (1H, d, *J* = 8.7 Hz, H-5) and 7.07 (1H, d, *J* = 8.7 Hz, H-6) for the 2,3,4-trihydroxyphenyl moiety; and δ_H_ 6.82 (1H, d, *J* = 8.7 Hz, H-5‴) and 7.48 (1H, d, *J* = 8.7 Hz, H-6‴) for the 2,3,4-trihydroxycinnamoy moiety, two *trans*-olefinic protons as AB-type signals at δ_H_ 6.35 (1H, d, *J* = 15.9 Hz, H-8‴) and 7.67 (1H, d, *J* = 15.9 Hz, H-7‴). In addition, two anomeric signals at δ_H_ 4.38 (1H, d, *J* = 7.9 Hz, H-1′)/δ_C_ 104.2 and δ_H_ 5.19 (1H, d, *J* = 1.6 Hz, H-1″)/δ_C_ 103.0, which were identified as β-d-glucopyranose and α-l-rhamnopyranose, respectively, glucose and rhamnose in **1**, were verified by TLC analysis after acid hydrolysis [2]. This above analytical data, combined with the NMR data, suggested that **1** is a phenylethyl glycoside [3]. The HMBC spectrum of **1** showed the key correlations between H-4′ and C-9‴, between H-3′ and C-1″ and between H-1′ and C-8, which suggested that the linkages of C-1′, C-3′ and C-4′ of glucose were directly connected to C-8 of the phenylethanol moiety, C-1″ of rhamnose and C-9‴ of the 2‴,3‴,4‴-trihydroxycinnamoyl moiety, respectively (Figure 2). Thus, the structure of **1** was elucidated as 1′-*O*-2,3,4-trihydroxy-phenylethoxy-*O*-α-l-rhamnopyranosyl-(1″→3′)-(4′-*O*-2‴,3‴,4‴-trihydroxycinnamoy)-β-D-glucopyranoside and amed marinoid J.

**Table 1:** ^1^H and ^13^C NMR data of **1** and **2** ^a^.

	Position	1		2	
		δ_C_, Mult	δ_H_ (*J* in Hz)	δ_C_, Mult	δ_H_ (*J* in Hz)
phenylethyl	1	115.9, C		121.4, C	
	2	147.6, C		148.6, C	
	3	130.6, C		103.9, CH	6.16 (s)
	4	156.8, C		143.7, C	
	5	116.0, CH	6.71 (d, 8.7)	143.5, C	
	6	131.0, CH	7.07 (d, 8.7)	110.1, CH	6.41 (s)
	7	36.3, CH_2_	2.81–2.84 (m)	34.6, CH_2_	2.83–2.87 (m)
	8	72.3, CH_2_	4.02–4.06 (m)	72.3, CH_2_	4.00–4.04 (m)
			3.70–3.74 (m)		3.71–3.75 (m)
glucosyl	1′	104.2, CH	4.38 (d, 7.9)	103.5, CH	4.78 (d, 7.3)
	2′	76.2, CH	3.38 (dd, 9.1, 7.9)	74.5, CH	3.40 (dd, 9.1, 7.3)
	3′	81.3, CH	3.81 (t, 9.1)	81.1, CH	3.84 (t, 9.1)
	4′	70.6, CH	4.91 (t, 9.1)	70.5, CH	4.94 (t, 9.1)
	5′	76.0, CH	3.52–3.57 (m)	76.1, CH	3.50–3.55 (m)
	6′	62.3, CH_2_	3.58–3.63 (m)	60.3, CH_2_	3.61–3.65 (m)
			3.49–3.54 (m)		3.52–3.56 (m)
rhamnosyl	1″	103.0, CH	5.19 (d, 1.6)	102.0, CH	4.58 (d, 1.6)
	2″	72.0, CH	3.91 (dd, 3.5, 1.6)	72.1, CH	3.92 (dd, 3.1, 1.2)
	3″	72.2, CH	3.56 (dd; 9.5, 3.5)	72.3, CH	3.58 (dd; 9.7, 3.1)
	4″	73.8, CH	3.28 (t, 9.5)	73.5, CH	3.25 (t, 9.7)
	5″	70.4, CH	3.54–3.58 (m)	70.6, CH	3.55–3.59 (m)
	6″	18.4, CH_3_	1.08 (d, 6.2)	18.2, CH_3_	1.05 (d, 6.2)
cinnamoyl in **1**	1‴	127.8, C		127.7, C	
caffeoyl in **2**	2‴	147.5, C		115.3, CH	7.00 (d, 1.5)
	3‴	133.4, C		143.6, C	
	4‴	161.5, C		145.6, C	
	5‴	117.7, CH	6.82 (d, 8.7)	119.5, CH	6.94 (d, 6.5)
	6‴	130.9, CH	7.48 (d, 8.7)	123.2, CH	6.60 (dd, 6.5, 1.5)
	7‴	147.8, CH	7.67 (d, 15.9)	144.5, CH	7.46 (d, 15.8)
	8‴	115.6, CH	6.35 (d, 15.9 )	115.3, CH	6.39 (d, 15.8 )
	9‴	168.3, C		169.2, C	

^a^ In CD_3_OD, 600 MHz for ^1^H and 150 MHz for ^13^C NMR.

Marinoid K (**2**), a yellow oil, has a molecular formula that was elucidated as C_29_H_36_O_16_ with the aid of the NMR and HR-ESI-MS data ([M + Na]^+^, *m*/*z*: 663.1898; calcd. for C_29_H_36_O_16_Na *m*/*z*: 663.1901). The ^1^H, ^13^C NMR (Table 1), COSY and HMQC spectra of **2** indicated that the presence of proton signals at δ_H_ 6.16 (1H, s, H-3) and 6.41 (1H, s, H-6) for the 2,4,5-trihydroxyphenyl moiety; and three proton signals at δ_H_ 7.00 (1H, d, *J* = 1.5 Hz, H-2‴), 6.94 (1H, d, *J* = 6.5 Hz, H-5‴) and 6.60 (1H, dd, *J* = 6.5, 1.5 Hz, H-6‴) for the caffeoyl moiety, two *trans*-olefinic protons as AB-type signals at δ_H_ 6.39 (1H, d, *J* = 15.8 Hz, H-8‴) and 7.46 (1H, d, *J* = 15.8 Hz, H-7‴). In addition, two anomeric signals at δ_H_ 4.78 (1H, d, *J* = 7.3 Hz, H-1′)/δ_C_ 103.5 and δ_H_ 4.58 (1H, d, *J* = 1.6 Hz, H-1″)/δ_C_ 102.0, which were identified as β-d-glucopyranose and α-l-rhamnopyranose, glucose and rhamnose in **2**, were verified by TLC analysis after acid hydrolysis. From these above analytical data, combined with the NMR data, we proposed that **2**, like **1**, is a phenylethyl glycoside [3]. The linkages of C-1′, C-3′ and C-4′ of glucose were directly connected to C-8 of the phenylethanol moiety, C-1″ of rhamnose and C-9‴ of the caffeoyl moiety, respectively, which was further confirmed by the key HMBC correlations between H-4′ and C-9‴, between H-3′ and C-1″ and between H-1′ and C-8 (Figure 2). The structure of **2** was thus elucidated to be 1′-*O*-2,4,5-trihydroxy-phenylethoxy-*O*-α-L-rhamnopyranosyl-(1″→3′)-(4′-*O*-caffeoyl)-β-d-glucopyranoside and named marinoid K.

Marinoid L (**3**), a yellow oil, has a molecular formula that was determined to be C_23_H_26_O_12_ by HRESIMS (found [M + H]^+^ at *m*/*z* 495.1501, calcd. for [M + H]^+^, 495.1503), as well as ^1^H and ^13^C data (Table 2). The ^1^H, ^13^C NMR, COSY and HMQC spectra of **3** displayed proton signals at δ_H_ 6.61 (1H, s, H-3) and 6.64 (1H, s, H-6) for the 2,4,5-trihydroxyphenyl moiety; and three proton signals at δ_H_ 7.03 (1H, d, *J* = 1.5 Hz, H-2″), 7.01 (1H, d, *J* = 7.5 Hz, H-5″) and 6.67 (1H, dd, *J* = 7.5, 1.5 Hz, H-6″) for the caffeoyl moiety, two *trans*-olefinic protons as AB-type signals at δ_H_ 6.30 (1H, d, *J* = 17.2 Hz, H-8″) and 7.56 (1H, d, *J* = 17.2 Hz, H-7″). NMR spectra also indicated the presence of a β-glucosyl group, *i.e.*, one anomeric carbon resonance at δ_C_ 103.1 (C-1′) and one anomeric proton at δ_H_ 4.34 (1H, d, *J* = 7.5 Hz, H-1′), which was identified as β-d-glucopyranose, glucose in **3**, and verified by TLC analysis after acid hydrolysis. The ^1^H and ^13^C NMR spectra (Table 2) are similar to those of calceolarioside A, except for the extra hydroxyl group on the phenylethyl moiety **3** (**3**: 2,4,5-trihydroxyphenyl, while calceolarioside A: 3,4-dihydroxyphenyl) [4]. The gross structure was further established by the aid of COSY and HMBC experiments (Figure 2). The key HMBC correlations between H-4′ and C-9″ and between H-1′ and C-8 suggested that the linkages of C-1′ and C-4′ of glucose were directly connected to C-8 of the phenylethyl moiety and C-9″ of caffeoyl moiety, respectively (Figure 2). Thus, the structure of **3** was elucidated to be 1′-*O*-2,4,5-trihydroxy-phenylethoxy-(4′-*O*-caffeoyl)-β-d-glucopyranoside and named marinoid L.

**Table 2:** ^1^H and ^13^C NMR data of **3** and **4** ^a^.

	Position		3		4
		δ_C_, Mult	δ_H_ (*J* in Hz)	δ_C_, Mult	δ_H_ (*J* in Hz)
phenylethyl in **3**	1	125.8, C		134.3, C	
cinnamoyl in **4**	2	155.2, C		127.9, CH	7.60 (d, 7.6)
	3	112.2, CH	6.61 (s)	128.6, CH	7.40-7.45 (m, overlap)
	4	144.4, C		130.1, CH	7.40-7.45 (m, overlap)
	5	144.2, C		128.6, CH	7.40-7.45 (m, overlap)
	6	115.1, CH	6.64 (s)	127.9, CH	7.60 (d, 7.6)
	7	35.1, CH_2_	2.78–2.82 (m)	145.1, CH	7.73 (d, 15.6)
	8	60.8, CH_2_	3.92–3.96 (m)	117.3, CH	6.59 (d, 15.6)
			3.68–3.72 (m)		
	9			166.7, C	
glucosyl	1′	103.1, CH	4.34 (d, 7.5)	105.2, CH	4.28 (d, 7.3)
	2′	74.0, CH	3.37 (dd, 9.1, 7.5)	74.6, CH	3.33 (dd, 9.1, 7.3)
	3′	76.5, CH	3.81 (t, 9.1)	82.4, CH	4.09 (t, 9.1)
	4′	70.3, CH	4.91 (t, 9.1)	70.6, CH	4.51 (t, 9.1)
	5′	73.6, CH	3.54–3.57 (m)	73.1, CH	3.76–3.80 (m)
	6′	63.2, CH_2_	3.58–3.63 (m)	63.8, CH_2_	3.70–3.74 (m)
			3.49–3.54 (m)		3.54–3.59 (m)
caffeoyl in **3**	1″	129.1, C		104.1, CH	4.41 (d, 7.6)
glucosyl in **4**	2″	114.7, CH	7.03 (d, 1.5)	73.1, CH	3.29 (dd, 9.1, 7.6)
	3″	145.1, C		77.7, CH	4.02 (t, 9.1)
	4″	147.8, C		70.4, CH	4.49 (t, 9.1)
	5″	115.1, CH	7.01 (d, 7.5)	71.7, CH	3.72–3.76 (m)
	6″	121.7, CH	6.67 (dd, 7.5, 1.5)	62.7, CH_2_	3.69–3.72 (m)
					3.55–3.59 (m)
	7″	145.6, CH	7.56 (d, 17.2)		
	8″	114.7, CH	6.30 (d, 17.2)		
	9″	167.7, C			

^a^ In CD_3_OD, 600 MHz for ^1^H and 150 MHz for ^13^C NMR.

Marinoid M (**4**), a yellowish oil, has a molecular formula identified as C_21_H_28_O_12_ from the HREIMS for the peak at *m*/*z* 495.1475 [M + Na]^+^ (calcd. for C_21_H_28_O_12_Na, 495.1473) and the NMR data. The ^1^H, ^13^C NMR (Table 2), COSY and HMQC spectra of **4** showed signals at δ_H_ 7.60 (2H, d, *J* = 7.6, H-2/H-6) and 7.40–7.45 (3H, m, H-3/H-4/H-5), which showed that the phenyl moiety in compound **4** is not substituted; in addition to two *trans*-olefinic protons as AB-type signals at δ_H_ 6.59 (1H, d, *J* = 15.6 Hz, H-8) and 7.73 (1H, d, *J* = 15.6 Hz, H-7). NMR spectra also indicated the presence of two β-glucosyl groups by two anomeric protons at δ_H_ 4.41 (1H, d, *J* = 7.6 Hz, H-1ʺ) and 4.28 (1H, d, *J* = 7.3 Hz, H-1ʹ) and two anomeric carbon resonances at δ_C_ 104.1 (C-1ʺ) and 105.2 (C-1ʹ), which were further confirmed by TLC analysis after acid hydrolysis. The gross structure was further established by the aid of COSY and HMBC experiments (Figure 2). The HMBC spectrum of **4** showed key correlations between H-1′ and C-9 and between H-3′ and C-1″, which confirmed the linkages of C-3′ and C-1′ of glucose directly connected to C-1″ of glucose and C-9 of the cinnamoyl moiety, respectively (Figure 2). Thus, the structure of **4** was elucidated to be β-d-glucosyl-(1″→3′)-(1′-*O*-cinnamoyl)-β-d-glucopyranoside and named marinoid M.

The cellular antioxidant activities of compounds **1**–**4** were measured using the cellular antioxidant assay (CAA) assay. The EC_50_ values of compounds **1**–**4** were 23.0 ± 0.71 μM, 36.2 ± 1.83 μM, 114.5 ± 0.40 μM and 247.8 ± 2.47 μM, respectively, of the same order of the positive control quercetin (EC_50_ = 11.0 ± 0.18 μM).

## 3. Experimental Section

### 3.1. General Experimental Procedures

UV spectra were recorded in MeOH on a Perkin-Elmer Lambda 35 UV-Vis spectrophotometer (Wellesley, MA, USA). The IR spectra were measured in KBr on a WQF-410 FT-IR spectrophotometer (Beifen-Ruili, Beijing, China). NMR spectra were recorded on a Bruker AV 600 MHz NMR spectrometer with TMS as an internal standard (Bruker, Bremen, Germany). HRESIMS data were obtained from Bruker Maxis mass spectrometer (Bruker, Bremen, Germany). A Waters-2695 HPLC system, using a Sunfire™ C_18_ column (150 × 10 mm i.d., 10 μm, Waters, Milford, MA, USA) coupled to a Waters 2998 photodiode array detector (Waters, Milford, MA, USA) was used. Optical rotation data were measured by a Perkin-Elmer Model 341 polarimeter (Wellesley, MA, USA). The silica gel GF_254_ used for TLC was supplied by the Qingdao Marine Chemical Factory, Qingdao, China. Spots were detected on TLC under UV light or by heating after spraying with 5% H_2_SO_4_ in EtOH. All solvent ratios are measured v/v.

### 3.2. Plant Material

The fruits of *A*. *marina* were collected from Beihai City, Guangxi, China, in September 2011. The specimen was identified by Hangqing Fan from Guangxi Mangrove Research Center, Guangxi Academy of Sciences. A voucher specimen (2011-GXAS-008) was deposited in Guangxi Key Laboratory of Marine Environmental Science, Guangxi Academy of Sciences, China.

### 3.3. Extraction and Isolation

The fruits of *A*. *marina* (35.4 kg) were exhaustively extracted in a large metal bowl (diameter 80 cm, volume 50 L) with EtOH–CH_2_Cl_2_ (2:1, 3 × 30 L) at 25 °C for 3 × 4 days. The solvent was evaporated *in vacuo* to afford a syrupy residue (935 g) that was suspended in distilled water (1.5 L) and fractionated successively with petroleum ether (3 × 2 L), ethyl acetate (3 × 2 L) and *n*-butanol (3 × 2 L). The ethyl acetate soluble portion (165 g) was subjected to column chromatography on silica gel, using CHCl_3_–Me_2_CO (from 10:0 to 0:10) as the eluent, giving eleven fractions (A–M). Fraction L was subjected to column chromatography to afford ten sub-fractions (L1–L10). Sub-fraction L3 was subjected to Sephadex LH-20 column chromatography with CHCl_3_–MeOH (1:1), then separated by HPLC, using the mixtures of MeOH–H_2_O (45:55) to yield **1** (2.5 mg). Sub-fraction L4 was separated by HPLC, using the mixtures of MeOH–H_2_O (from 5:95 to 60:40) to yield **3** (4.0 mg) and **2** (3.1 mg), respectively. Fraction D was further subjected to column chromatography to afford four sub-fractions (D1–D4); sub-fraction D4 was separated by HPLC, using mixtures of MeOH-H_2_O (5:95) to yield **4** (2.2 mg).

Marinoid J (**1**): Yellow oil; 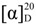 −24.7° (*c* 0.74, MeOH); UV (MeOH) λ_max_ (log ε) nm: 214 (4.12), 246 (4.00), 291 (4.12), 331 (3.75); IR (KBr) ν_max_ 3381, 1691, 1621 and 1602 cm^−1^; ^1^H (CD_3_OD, 600 MHz) and ^13^C (CD_3_OD, 150 MHz) NMR; see Table 1; HRESIMS *m*/*z* 657.2029 (calcd. for C_29_H_36_O_17_ + H, 657.2031).

Marinoid K (**2**): Yellow oil; 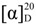 −35.5° (*c* 0.39, MeOH); UV (MeOH) λ_max_ (log ε) nm: 215 (3.75), 241 (3.97), 289 (4.01), 332 (3.67); IR (KBr) ν_max_ 3386, 1693, 1622 and 1600 cm^−1^; ^1^H (CD_3_OD, 600 MHz) and ^13^C (CD_3_OD, 150 MHz) NMR; see Table 1; HRESIMS: *m*/*z* 663.1898 (calcd. for C_29_H_36_O_16_ + Na, 663.1901).

Marinoid L (**3**): Yellow oil; 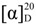 −32.2° (*c* 0.46, MeOH); UV (MeOH) λ_max_ (log ε) nm: 214 (4.09), 245 (4.01), 290 (4.12), 332 (3.23); IR (KBr) ν_max_ 3392, 1693, 1624 and 1600 cm^−1^; ^1^H (CD_3_OD, 600 MHz) and ^13^C (CD_3_OD, 150 MHz) NMR; see Table 2; HRESIMS: *m*/*z* 495.1501 (calcd. for C_23_H_26_O_12_ + H, 495.1503).

Marinoid M (**4**): Yellow oil; 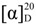 +13.2° (*c* 0.57, MeOH); UV (MeOH) λ_max_ (log ε) nm: 211 (3.98), 245 (3.91), 289 (4.10); IR (KBr) ν_max_ 3402, 1690, 1620 and 1602 cm^−1^; ^1^H (CD_3_OD, 600 MHz) and ^13^C (CD_3_OD, 150 MHz) NMR; see Table 2; HRESIMS: *m*/*z* 495.1475 (calcd. for C_21_H_28_O_12_ + Na, 495.1473).

### 3.4. Acid Hydrolysis of 1–4

Acid hydrolysis of **1**−**4** was carried out according to the reported method [2].

### 3.5. Cellular Antioxidant Assay

The cellular antioxidant activity (CAA) was carried out following the literature method [5].

## 4. Conclusions

Three new phenylethyl glycosides and a new cinnamoyl glycoside were isolated from the fruits of mangrove *A*. *marina* and identified. The CAA is a new approach to quantify antioxidant activity under physiological conditions when compared to chemical antioxidant activity assays [5]. The CAA assay has only recently been used in the marine natural product field [1]. Using this assay, compounds **2** and **3** showed relevant antioxidant activity comparable to the control, quercetin.
